# Engineered photoproteins that give rise to photosynthetically-incompetent bacteria are effective as photovoltaic materials for biohybrid photoelectrochemical cells

**DOI:** 10.1039/c7fd00190h

**Published:** 2017-11-01

**Authors:** Juntai Liu, Vincent M. Friebe, David J. K. Swainsbury, Lucy I. Crouch, David A. Szabo, Raoul N. Frese, Michael R. Jones

**Affiliations:** a School of Biochemistry , University of Bristol , Medical Sciences Building, University Walk , Bristol BS8 1TD , UK . Email: m.r.jones@bristol.ac.uk; b Department of Physics and Astronomy , LaserLaB Amsterdam , VU University Amsterdam , De Boelelaan 1081, 1081 HV , Amsterdam , The Netherlands

## Abstract

Reaction centre/light harvesting proteins such as the RCLH1X complex from *Rhodobacter sphaeroides* carry out highly quantum-efficient conversion of solar energy through ultrafast energy transfer and charge separation, and these pigment-proteins have been incorporated into biohybrid photoelectrochemical cells for a variety of applications. In this work we demonstrate that, despite not being able to support normal photosynthetic growth of *Rhodobacter sphaeroides*, an engineered variant of this RCLH1X complex lacking the PufX protein and with an enlarged light harvesting antenna is unimpaired in its capacity for photocurrent generation in two types of bio-photoelectrochemical cells. Removal of PufX also did not impair the ability of the RCLH1 complex to act as an acceptor of energy from synthetic light harvesting quantum dots. Unexpectedly, the removal of PufX led to a marked improvement in the overall stability of the RCLH1 complex under heat stress. We conclude that PufX-deficient RCLH1 complexes are fully functional in solar energy conversion in a device setting and that their enhanced structural stability could make them a preferred choice over their native PufX-containing counterpart. Our findings on the competence of RCLH1 complexes for light energy conversion *in vitro* are discussed with reference to the reason why these PufX-deficient proteins are not capable of light energy conversion *in vivo*.

## Abstract 2

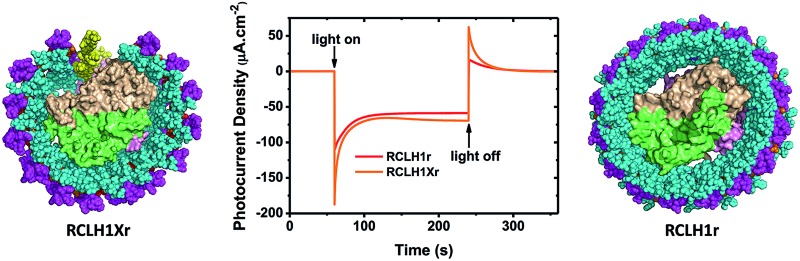
We address concerns that PufX-deficient RCLH1 complexes from photosynthetically-incompetent bacteria may not be suitable as photovoltaic materials for incorporation into biohybrid photoelectrochemical cells.

## 1. Introduction

In the quest to develop new sustainable materials for solar energy conversion, there has been increasing interest in combining pigment-proteins from photosynthetic organisms with man-made materials in biohybrid device architectures.^1^–^9^ The primary events in light harvesting (LH) and reaction center (RC) proteins transduce solar energy with a near to unity quantum efficiency (charges separated per photon absorbed).^10^–^15^ In layman’s terms, natural photosystems act as solar batteries, using sunlight to create a potential difference across the photosynthetic membrane. Connection of additional electron transfer “wires” to the positive and negative “terminals” of these solar batteries powers subsequent biological processes. A variety of RC or combined RC/LH proteins have been incorporated into device architectures in order to harness charge separation for photocurrent generation and other applications such as sensing.^1^–^9^ Much of this work has employed the RC from *Rhodobacter* (*Rba.*) *sphaeroides*, a pigment-protein that has informed much of our understanding of solar energy conversion in photosynthetic RCs.^16^–^20^

**Fig. 1:**
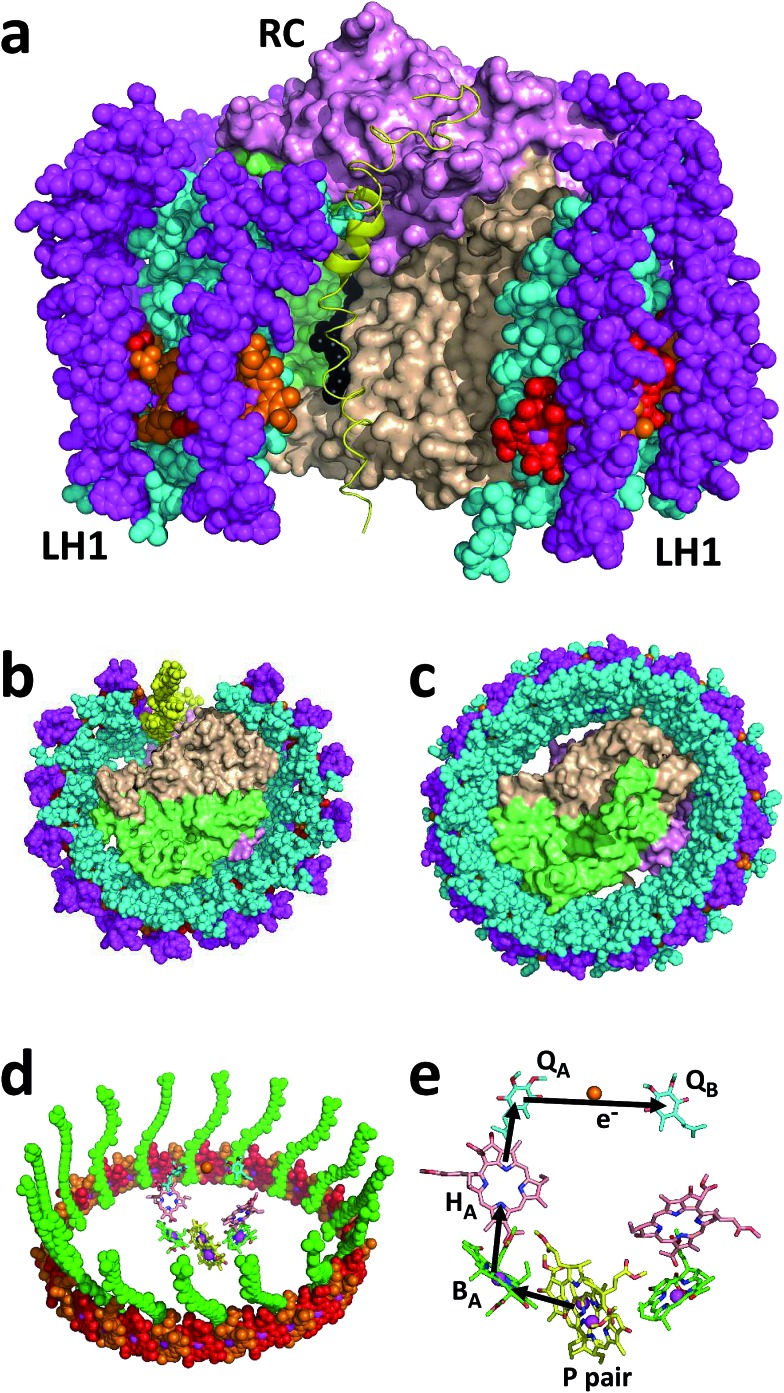
Structure and mechanism of the *Rba. sphaeroides* RCLH1X and RCLH1 complexes. (a) A view approximately in the plane of the photosynthetic membrane of a RCLH1X monomer. The colour/representation coding is: pink surface – RC H-polypeptide, lime-green surface – RC L-polypeptide, beige surface – RC M-polypeptide, yellow ribbon – PufX, cyan spheres – LH1 α-polypeptides, magenta spheres – LH1 β-polypeptides, red/orange spheres – alternating LH1 BChls with the central Mg shown in magenta, black spheres – side chain of the RC Q_B_ ubiquinone-10. (b) A view from the periplasmic side of the membrane of the *Rba. sphaeroides* RCLH1X complex. (c) The same view of the *T. tepidum* RCLH1 complex. (d) The 32 BChls and 16 carotenoids of *T. tepidum* LH1 (spheres) arranged around the cofactors of the central RC (sticks). The colour coding for the LH1 cofactors is: red/orange spheres – alternating LH1 BChls with the central Mg shown in magenta, green spheres – carotenoids. The colour coding for the RC cofactors is: yellow carbons – P BChls, green carbons – monomeric BChls, pink carbons – bacteriopheophytins, cyan carbons – ubiquinones, brown spheres – iron atoms, magenta spheres – BChl Mg atoms. (e) Cofactors of the *Rba. sphaeroides* RC and the route of electron transfer. The cofactor representation is the same as for (d). For clarity, cofactor hydrocarbon side chains are not shown.

Purple bacterial RCs are typically enclosed within a hollow cylinder of the LH1 pigment-protein, forming the so-called RC–LH1 core complex (Fig. 1(a)).^21^–^23^ In many species the light harvesting capacity of the photosystem is augmented by a peripheral LH2 antenna.^24^ The RC, LH1 and LH2 pigment-proteins can assemble independently of one another in *Rba. sphaeroides*, and because the photosystem components are assembled during respiratory growth at moderate oxygen levels in the dark in this bacterium, they can be expressed in any combination.^25^ This modularity is very useful if the bacterium is being used as a “factory” for the production of tailored proteins, enabling tuning of the relative contributions of light harvesting and charge separation to suit a particular device setting or application. The pigmentation of the photosystem can also be varied by selection of spontaneously-arising strains that have a dysfunction in one or more of the enzymes of the carotenoid synthesis pathway. The best known of these are “green strains” where the native red/brown carotenoids, spheroidene and spheroidenone, are replaced by their precursor neurosporene.^26^,^27^ Red and green variants of the RC–LH1 complex were recently used in a comparison of photocurrent generation by bio-photoelectrochemical cells in mixed or tandem configurations, exploiting natural variation.^28^ The carotenoids incorporated into the *Rba. sphaeroides* photosystem can also be diversified by heterologous expression of non-native genes and pathways.^29^,^30^

The *Rba. sphaeroides* RC–LH1 complex includes a PufX polypeptide that breaks the continuity of the LH1 cylinder (see ref. 31 for a review), and its location was revealed in a 7.8 Å resolution X-ray crystal structure of the “RCLH1X complex” (Fig. 1(a), yellow ribbon).^22^ Each LH1 comprises 14 pairs of α and β membrane-spanning polypeptides that scaffold 28 carotenoids and 28 BChls as LH pigments, and when viewed perpendicular to the plane of the membrane forms a C-shaped antenna with a gap held open by PufX (Fig. 1(b), left). In addition, these RCLH1X proteins assemble into dimers around a two-fold symmetry axis, such that two RCs are surrounded by a continuous S-shaped LH1 antenna.^21^,^22^,^32^–^34^ If the gene encoding PufX is deleted, the resulting “RCLH1 complexes” are uniformly monomeric,^35^ with a single RC encased in a complete LH1 cylinder^33^,^36^,^37^ comprising 16 pairs of α and β polypeptides, 32 carotenoids and 32 BChls. Removal of PufX therefore increases the number of LH pigments servicing each RC and simplifies the composition of the prepared protein to one of uniform monomers. The X-ray crystal structure of the PufX-deficient *Rba. sphaeroides* complex is not available, but Fig. 1(c) shows the 3.0 Å resolution X-ray crystal structure of the RCLH1 complex from *Thermochromatium* (*T.*) *tepidum*.^23^ This lacks PufX and has a closed 16-member LH1 ring around the RC, and so serves as a useful model for the RCLH1 complex from *Rba. sphaeroides*. This *T. tepidum* structure includes one carotenoid per LH1 subunit, and so enables the arrangement of pigments around the RC cofactors to be visualised (Fig. 1(d)). *Rba. sphaeroides* RCLH1(X) complexes contain two carotenoids per LH1 subunit,^38^ but these were not resolved in the available X-ray crystal structure.^22^ These rings of BChl and carotenoid pigments harvest light energy and “feed” the resulting excited state to the RC electron transfer chain.

Arrival of the excited state at a pair of bacteriochlorophyll (BChl) electron transfer cofactors (*P*) in the RC triggers a four-step charge separation to a mobile ubiquinone-10 (Q_10_) located on the opposite side of the membrane at the so-called Q_B_ site (Fig. 1(e)).^16^–^20^ This transfer occurs *via* a BChl (B_A_), bacteriopheophytin (BPhe–H_A_) and an immobile Q_10_ (Q_A_) (Fig. 1(e)), with the system evolving along the sequence P* → P^+^B_A_^–^ → P^+^H_A_^–^ → P^+^Q_A_^–^ → P^+^Q_B_^–^. The final radical pair is stabilised by the reduction of P^+^ by a diffusible c-type cytochrome (cyt) whose role is to shuttle electrons to the RC from a partner cyt *bc*_1_ complex. A second light-powered charge separation elicits double reduction and double protonation of Q_B_ to produce a ubiquinol (Q_10_H_2_).^39^,^40^ This dissociates from the RC and passes through the surrounding LH1 protein into the membrane interior in order to supply electrons to the cyt *bc*_1_ complex. Several studies of the photovoltaic capacity of the *Rba. sphaeroides* RC or RCLH1X complexes in a device setting have recapitulated the interactions of the oxidising and reducing “terminals” of the RC with cyt *c* and ubiquinone. Cyt *c* has been used to “wire” RCs to a working electrode, thus enabling the reduction of photogenerated P^+^ by a cathodic current (see ref. 41 and the references therein), and a water-soluble analogue of Q_10_, ubiquinone-0 (Q_0_), has been used to mediate charge flow from the Q_B_ terminal of the RC to a counter electrode (see ref. 42 and the references therein).

Despite its enhanced light harvesting capacity and its simplified, exclusively monomeric architecture, a point of concern over the use of PufX-deleted RCLH1 complexes for device applications is that this modification renders *Rba. sphaeroides* incapable of growth under standard anoxic, illuminated conditions.^43^–^47^ This impairment is often portrayed in terms of the enlarged LH1 ring blocking the escape of Q_10_H_2_ from the RCLH1 complex and blocking its replacement by oxidised Q_10_ from the intramembrane quinone pool, diffusional processes that are normally facilitated by PufX keeping the LH1 ring open (see ref. 38 for a recent discussion). In the representation of an RCLH1X monomer in Fig. 1(a) the atoms coloured in black, visible behind PufX (yellow), are part of the hydrocarbon side chain of the Q_B_ ubiquinone; the quinone head-group is above these, buried in a binding pocket in the interior of the RC. It is easy to conceptualise that the replacement of PufX by an extra section of LH1 pigment-protein could fill the gap maintained by PufX and prevent quinone diffusion. Regardless of whether this “blockage mechanism” is correct (see Section 3.7), the fact that strains of *Rba. sphaeroides* with PufX-deficient RCLH1 complexes are incapable of photosynthetic growth raises obvious concerns over their suitability as a material for photocurrent generation. The fragmentary experimental data published to date supports such concerns, as bio-photoelectrochemical cells fabricated using RCLH1 complexes have been reported to generate markedly lower steady-state photocurrent densities, in the range of 0.15 to 8.6 μA cm^–2^,^28^,^48^–^50^ than is the case for PufX-containing RCLH1X complexes produced from equivalent strains and by equivalent purification procedures, where current densities of up to 166 μA cm^–2^ have been described.^51^

In this report, we profile the types of RCLH1X complex that can be isolated from strains of *Rba. sphaeroides* with different types of carotenoid, examine how the removal of PufX affects these profiles, and compare the abilities of the RCLH1X and RCLH1 complexes to support a photocurrent. We also compare the abilities of the two complexes to interact with synthetic antenna nanocrystals, and their structural stabilities under stress conditions. We discuss why the removal of PufX produces a photosynthesis-minus phenotype, and also discuss the validity of using the resulting RCLH1 complexes for photocurrent generation in biohybrid devices.

## 2. Experimental

### 2.1 Biological materials

*Rba. sphaeroides* strains with either red or green carotenoid expressing RCLH1X and RCLH1 complexes were constructed as described previously^28^,^51^ and were named as RCLH1Xr, RCLH1r, RCLH1Xg or RCLH1g. Bacteria were grown either under dark/semi-aerobic conditions in an unilluminated orbital incubator operating at 34 °C and 180 rpm,^52^ or in completely filled 500 mL medical flat bottles in a glass circulating water bath at 34 °C that was illuminated with four 100 Watt incandescent light bulbs. The RCLH1(X) and RC complexes were purified using a His_10_-tag on the PufM polypeptide of the RC component as described elsewhere.^51^,^53^

### 2.2 Protein profiling on sucrose density gradients

Intracytoplasmic membranes prepared using a French pressure cell^52^ were suspended in 20 mM HEPES (pH 8) to a concentration equivalent to an absorbance of 60 at the maximum of the LH1 Q_*y*_ absorbance band, and DDM was added to 4% final concentration (w/v). After incubation on ice for 30 min in the dark, membrane debris was removed through 1 hour of centrifugation in a TLA 100 rotor at 78 100*g* and 4 °C. Sucrose density gradients comprising 2 mL steps of 20, 21.25, 22.5, 23.75 and 25% (w/v) sucrose in 20 mM HEPES (pH 8)/0.04% DDM were prepared in transparent ultracentrifuge tubes. Each gradient was loaded with a 150 μL aliquot of solubilised protein at an absorbance of 25 at 875 nm. The loaded gradients were centrifuged for 20 hours in a Sorvall TH-641 swing-out rotor at 180 000*g* and 4 °C.

### 2.3 Photochronoamperometry of biohybrid protein/silver electrodes

Nanostructured silver electrodes were prepared from 3 mm diameter planar silver electrodes (Metrohm Autolab) as described previously.^51^ RCLH1X, RCLH1 or RC proteins at a concentration of 30 μM were drop-casted onto the prepared electrodes in the dark at 4 °C for 15 minutes and unbound protein was removed by repeated mechanically-controlled dipping in 20 mM Tris (pH 8) at 4 °C. Protein-coated electrodes were mounted in a photoelectrochemical cell fitted with a platinum counter electrode and a Ag/AgCl/3 M KCl reference electrode, and immersed in an electrolyte solution comprising 20 mM Tris (pH 8) supplemented with 200 μM cyt *c* and 1.0 mM ubiquinone-0 (Q_0_).^51^ Photocurrents were measured at room temperature and a bias potential of –100 mV *vs.* Ag/AgCl under the control of a PGSTAT128N potentiostat (Metrohm Autolab). Illumination was supplied by an 870 nm LED (Roithner Lasertechnik) with an irradiance of 46 mW cm^–2^ at the electrode surface.

### 2.4 Photochronoamperometry of biohybrid protein/gold electrodes

Planar gold electrodes with a 2 mm diameter (CHI Instruments) were prepared as described previously.^53^ RCLH1X or RCLH1 proteins at a concentration of 30 μM were drop-casted onto cleaned electrodes in the dark at 4 °C for 60 minutes and unbound protein was removed by repeated mechanically-controlled dipping in 20 mM Tris (pH 8) at 4 °C. Protein-coated planar gold electrodes were mounted in a photoelectrochemical cell as described above for the protein/silver electrodes, but in an electrolyte solution comprising 20 mM Tris (pH 8)/20 μM cyt *c*/100 μM UQ_0_.^42^ Photocurrents were measured as for the protein/silver electrodes.

### 2.5 Titrations of quantum dot emission

Water-soluble 6.5 nm-diameter CdTe QDs were purchased from PlasmaChem GmbH (Berlin, Germany). Solutions of QDs and RCLH1Xg or RCLH1g complexes in 20 mM Tris (pH 8)/0.04% DDM were mixed to give protein : QD ratios that varied between 0 and 10 : 1. The emission in response to 515 nm or 645 nm excitation was recorded using a Cary Eclipse fluorescence spectrophotometer (Agilent).

### 2.6 Sucrose pull-down assays

Protein/QD conjugates were separated from free proteins by centrifugation on sucrose density gradients comprising 5 mL 60% sucrose and 5 mL 30% sucrose, both (w/v) in 20 mM Tris (pH 8)/0.04% DDM. Protein-only or QD/protein mixed samples were added in volumes of 400 μL and overlaid with 1 mL of 20 mM Tris (pH 8.0)/0.04% DDM. The protein concentration was fixed at 250 nM and the QD concentration was varied in order to achieve the indicated QD/protein ratios. Loaded tubes were centrifuged for 16 h at 247 104*g* and 4 °C in a TH-641 swinging bucket rotor. The gradients were deconstructed by piercing the bottom of the tube and collecting 11 × 1 mL fractions dropwise, and the pigment-protein content of each fraction was determined by absorbance spectroscopy.

### 2.7 Assays of thermal stability

Assays were carried out using solutions of RCLH1(X) complexes in completely filled, stoppered quartz cuvettes incubated at the desired temperature in a water-thermostatted multi-cell holder linked to a circulating water bath and mounted in a Cary 60 scanning spectrophotometer (Agilent). The temperature in the cuvette was monitored using a Fluke 51 thermometer.

### 2.8 Molecular models

Images of the protein structure were produced using the PyMOL molecular graphics system (Schrödinger, LLC) using the Protein Data Bank entries 4V9G for the *Rba. sphaeroides* RCLH1X complex,^22^; 3WMM for the *T. tepidum* RCLH1 complex^23^ and ; 1HRC for the cyt *c* from *Equus caballus*.^54^

## 3. Results and discussion

### 3.1 The RC–LH1 complexes obtainable from *Rba. sphaeroides*

**Fig. 2:**
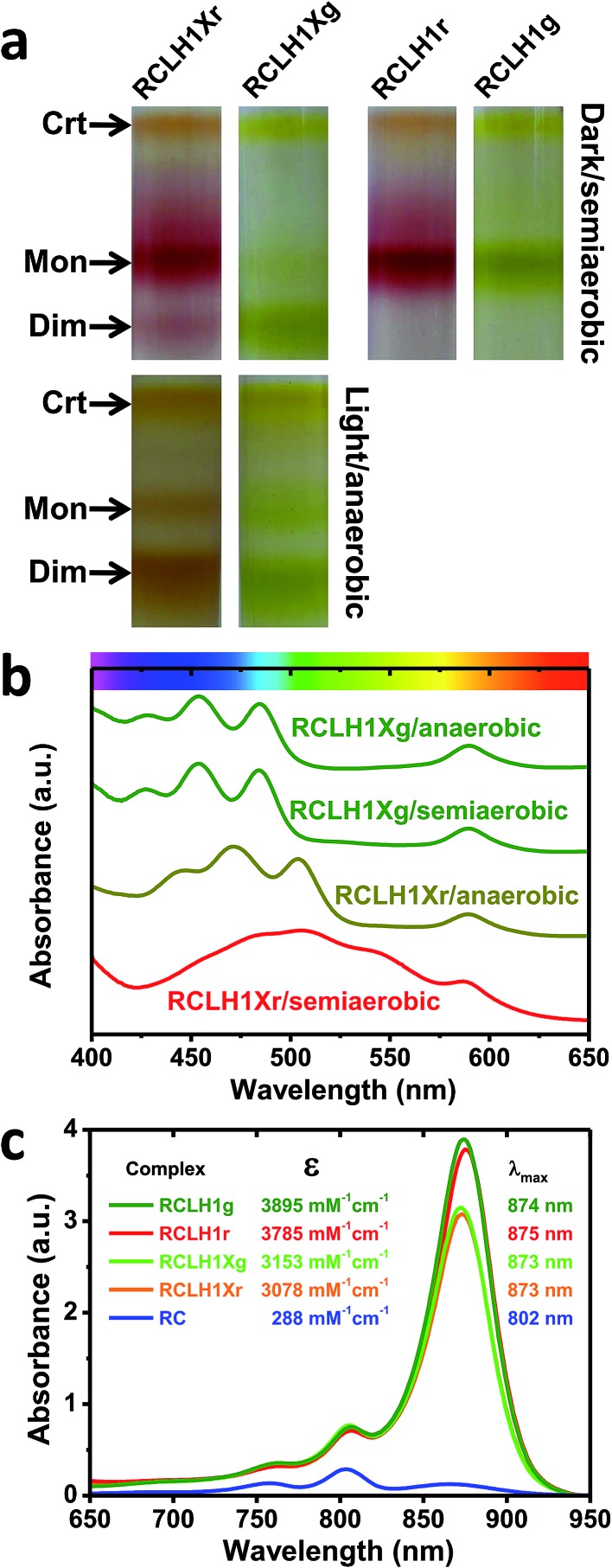
A variety of RCLH1 complexes from *Rba. sphaeroides*. (a) Bands formed by pigment-proteins separated on sucrose density gradients, (b) visible region absorbance spectra for the RCLH1Xr and RCLH1Xg complexes, (c) molar absorption coefficients for the purified RCLH1(X) or RC complexes and near-IR absorbance spectra for 1 μM solutions.

The pigment-proteins that can be extracted from LH2-deficient strains of *Rba. sphaeroides* are profiled in Fig. 2(a). For strain RCLH1Xr, fractionation of DDM-extracted proteins on a five-step sucrose density gradient produced three coloured bands (Fig. 2(a), left) corresponding to free carotenoid (arrow Crt), RCLH1X monomers (arrow Mon) and RCLH1X dimers (arrow Dim). The colour of the protein bands was determined by the type of carotenoid present, this being spheroidenone when strain RCLH1Xr is grown in the presence of oxygen (Fig. 2(a), top) and spheroidene when strain RCLH1Xr is grown in the absence of oxygen (Fig. 2(a), bottom). The absorbance of spheroidene in these proteins largely cuts off before the yellow/orange region of the visible spectrum (Fig. 2(b), brown trace), hence the colour change from the red colour conferred by spheroidenone (Fig. 2(a), top left) to the brown colour conferred by spheroidene (Fig. 2(a), bottom left).

The same pattern was seen for strain RCLH1Xg (Fig. 2(a), column 2) which synthesises neurosporene and its hydroxy and methoxy derivatives.^25^ The absorbance of the neurosporene family carotenoids largely cuts off before the green region of the visible spectrum (Fig. 2(b), green spectra), and the absorbance line shape is not variable with the presence/absence of oxygen.

The architectures of the isolated complexes depended on the type of carotenoid present. With strain RCLH1Xg, and strain RCLH1Xr grown in the absence of oxygen, the percentage of RCLH1X complexes in the dimer form (Fig. 2(a)) was much greater than that in the monomeric form. In contrast, when strain RCLH1Xr was grown in the dark in the presence of oxygen, causing the incorporation of spheroidenone, most of the extracted RCLH1X complexes were monomeric (Fig. 2(a), top left). As expected, the removal of PufX to produce a pair of otherwise equivalent strains, denoted as RCLH1r and RCLH1g, resulted in the complete loss of the dimer form in cells grown under dark/semiaerobic conditions (Fig. 2(a), top right). These variations in the relative amounts of the monomeric and dimeric complexes, dependent on the growth conditions, carotenoid type and presence of PufX, were also seen in the strains expressing LH2 (data not shown).

### 3.2 Absorbance changes caused by the removal of PufX

RCLH1(X) complexes were purified from strains expressing red or green carotenoids grown under dark/semiaerobic conditions (see Experimental). Analysis using sucrose density gradient ultracentrifugation showed that the purified RCLH1r and RCLH1g complexes were exclusively monomeric, as were the RCLH1Xr complexes (data not shown). A minority of the RCLH1Xg complexes were dimeric, but were not separated from the major monomeric population before further analysis.

Molar absorption coefficients for the four variants were estimated by recording absorbance spectra for aliquots of the concentrated protein diluted in 20 mM Tris (pH 8.0)/0.04% DDM and also recording absorbance spectra for aliquots diluted in 7 : 2 acetone : methanol. The concentration of extracted BChl in the latter was determined using a molar absorption coefficient of 65.3 mM^–1^ cm^–1^ for BChl *a* in this solvent.^55^ A molar absorption coefficient for each protein was then calculated at the maximum of the LH1 Q_*y*_ absorbance band between 873 nm and 875 nm based on the assumption that a RCLH1X complex has 32 BChl *a* and a RCLH1 complex has 36 BChl *a*.

The determined values are shown in Fig. 2(c), along with the calculated absorbance spectra for each complex at a concentration of 1 μM and purified RCs at the same concentration. For each carotenoid type, PufX removal caused an increase in intensity and a 1–2 nm red-shift of the LH1 Q_*y*_ absorbance band, as well as an increase in the carotenoid region (not shown), which is in accordance with the expectation that the PufX-deficient version will contain four extra BChl and four extra carotenoids per RC. The estimated molar absorption coefficient was 1.23-fold larger for the RCLH1r complexes compared to the RCLH1Xr complexes, and 1.24-fold larger for the green counterparts.

### 3.3 Photocurrents from the RCLH1X, RCLH1 and RC complexes

**Fig. 3:**
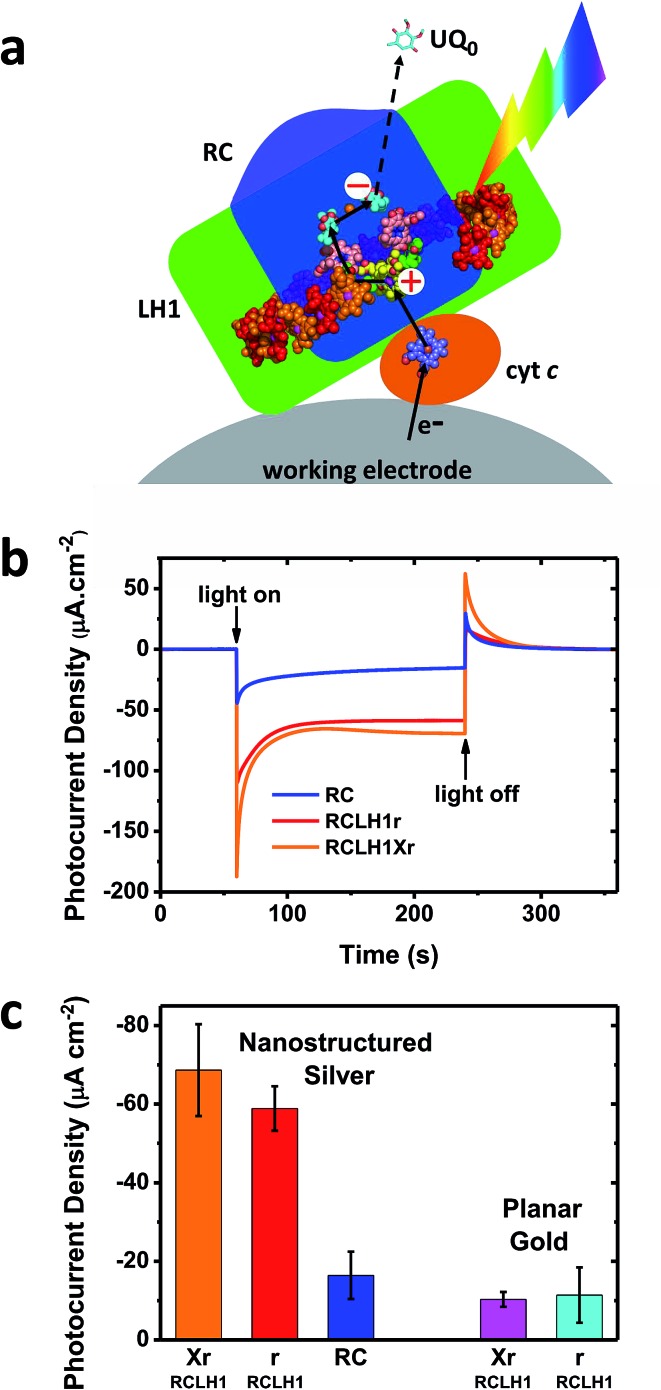
Photocurrents from the RCLH1Xr, RCLH1r and RC complexes interfaced with metal electrodes. (a) Photoexcitation of the BChls (alternating red/orange) and carotenoids (not shown) of the LH1 antenna (green domain) produces charge separation in the RC (blue domain). The RC is re-reduced by electrons from the working electrode in a process mediated by cyt *c* (orange protein). The transfer of electrons to the counter electrode is mediated by 1 mM Q_0_. For a representation of the RCLH1 components, see Fig. 1. The haem of cyt *c* is shown as slate-blue spheres with a brown Fe sphere. (b) Photocurrent transients in response to 180 seconds of illumination of the RCLH1Xr, RCLH1r or RC complexes adhered to nanostructured silver working electrodes. (c) A comparison of the steady state photocurrent densities for proteins adhered to nanostructured silver or planar gold working electrodes.

The capacity for photocurrent generation was tested using purified RCLH1Xr or RCLH1r complexes drop-casted onto a nanostructured silver working electrode. This was immersed in a buffer containing 1 mM Q_0_ and 200 μM cyt *c*.^51^ Illumination of the working electrode produced a cathodic photocurrent, with the underlying mechanism investigated in-depth in cells with RC complexes^41^,^42^ which is summarised in Fig. 3(a). Cyt *c* facilitates current flow from the working electrode to the protein,^41^ with Q_0_ carrying electrons to the counter electrode in a process that requires full cycles of two-electron/two-proton chemistry at the Q_B_ site.^42^ An additional feature of the RCLH1(X) complexes used in the present work is the expected presence of up to ∼25 molecules of endogenous Q_10_ per monomer.^56^ The role these play in enabling electron flux from the RC into solution is the subject of ongoing investigation, but it is clearly established that Q_0_H_2_ is the actual charge carrier to the counter electrode, as Q_10_ in solution has been shown to be unable to support a photocurrent.^42^

**Fig. 4:**
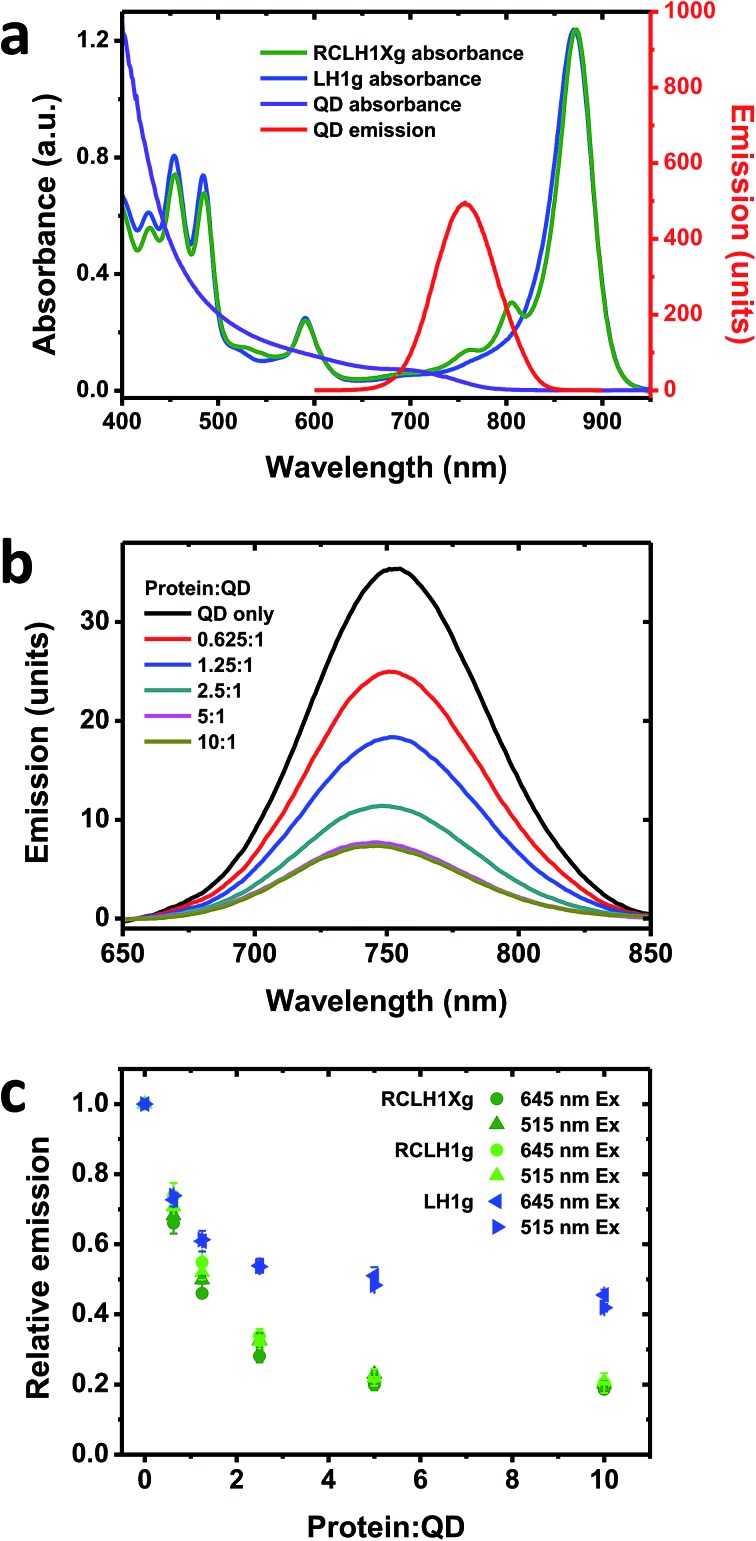
Quenching of QD emission by the RCLH1Xg or RCLH1g complexes. (a) Absorbance spectra of the RCLH1Xg and LH1g complexes, and absorbance and emission spectra of 6.5 nm-diameter water soluble CdTe QDs, (b) quenching of emission of 50 nM QDs by 31.25–500 nM RCLH1Xg complexes, with excitation at 515 nm, (c) decay of relative QD emission as a function of protein : QD ratio, using data collected at two excitation wavelengths. The data points are means with standard deviation (*n* = 3).

All transients showed an initial spike of cathodic photocurrent following light-on that stabilised over a minute or so to a lower steady-state level (Fig. 4(b)). This initial decline has been attributed to a limitation of current output due to diffusion-limited mass transport of the Q_0_ electrolyte.^51^ When the light was switched off the recombination of the accumulated Q_0_H_2_ product with the working electrode produced a transient spike of anodic current.

For the working electrodes with adhered RCLH1Xr complexes, the average steady-state photocurrent density determined over the last 80 s of the illumination period was approximately –69 μA cm^–2^ (Fig. 3(b) and (c)). This was much larger than the average density of –16 μA cm^–2^ determined for the RC complexes lacking the LH1 antenna adhered to the same nanostructured silver electrodes in an otherwise identical measuring system (Fig. 3(b) and (c)), thus demonstrating the benefit of using a combined LH/RC protein for photocurrent generation rather than a “naked” RC.

Despite being isolated from a strain of *Rba. sphaeroides* that is not capable of photosynthetic growth, PufX-deficient RCLH1r complexes generated a photocurrent that was not significantly different to that seen for native RCLH1Xr complexes (Fig. 3(b) and (c)). A similar result was obtained by equivalent measurements using planar gold as the working electrode (Fig. 3(c), right). The absolute photocurrent densities were lower with the planar gold electrodes than with nanostructured silver, which is in accordance with the lower surface area for protein adhesion and a lack of plasmonic enhancement provided by the nanostructured silver surface.^51^

### 3.4 Energy transfer from the synthetic antenna structures to the RCLH1X and RCLH1 complexes

One drawback of using natural proteins in a photoelectrochemical cell is that low currents are produced at excitation wavelengths where their LH pigments have weak absorbance. As a result, there is growing interest in the augmentation of native absorbance across the UV/visible/near-IR spectrum and the fabrication of hybrid photosystems that combine natural proteins with synthetic light harvesting materials. One option is to modify the absorbance of purple bacterial RCs with quantum dots (QDs), nanocrystalline materials whose absorbance and emission properties are dependent on their composition and size.^57^ In addition to their broad visible region absorbance, these exhibit strong absorbance in the UV region which may be of interest with regard to photoprotection and the stability of biohybrid systems.

As RCLH1(X) complexes appear to be more effective photovoltaic materials than naked RCs, in this work we looked at their ability to accept energy from QDs and any effects of the removal of PufX. The QDs used were 6.5 nm-diameter nanostructures comprising cadmium telluride (CdTe) that were rendered water-soluble by coating with carboxyl terminated groups. These exhibit a broad absorbance that commences at around 800 nm and extends across the visible region, rising strongly in the blue and UV regions (Fig. 4(a), purple). They possess a symmetrical emission band (Fig. 4(a), red) that overlaps with the RC Q_*y*_ absorbance bands at 760 and 800 nm and the blue edge of the dominant LH1 Q_*y*_ absorbance band, centred at 873 nm. For this work, complexes with green carotenoids were used, as these exhibit lower absorbance in the 500–650 nm region than is the case for complexes with red carotenoids (see Fig. 2(b)). This creates a better window for QD excitation at 515 nm, in-between the strong carotenoid absorbance below 505 nm and the BChl Q_*x*_ band between 565 and 625 nm (Fig. 4(a), green), in addition to excitation at 645 nm where absorbance of the pigment-protein is at a minimum.

To look for energy transfer a fixed concentration of 50 nM QDs was mixed with up to a 10 : 1 molar ratio of RCLH1Xg or RCLH1g protein. A concentration-dependent drop in QD emission occurred that levelled off at ∼80% quenching above a molar ratio of 5 : 1 (Fig. 4(b)). The QD emission maximum also blue-shifted by several nm, likely reflecting stronger quenching on the red side of the emission band due to spectral overlap with the protein being much larger on the red side of the band than on the blue side.

Repeat titrations showed that there was no dependence of the extent of quenching on the excitation wavelength (compare the circles and triangles in Fig. 4(c)). As the absorbance of the protein was around four-fold stronger at 515 nm than at 645 nm, this suggested that the drop in QD emission upon increasing the protein concentration was not due to shading by the pigments in the added protein (the protein and QD concentrations were kept low to avoid this, with the protein absorbance always below 0.05 absorbance units at 645 nm and 0.2 at 515 nm). In addition, the spectra of the most strongly quenched samples did not show any evidence of the appearance of inverse band structures that would indicate that the drop in QD emission was being affected by the reabsorption of light emitted by the QDs by BChl (such artefacts, negative bands at 760 nm and to a lesser extent at 800 nm, were apparent in titrations carried out at fifty-fold higher QD and protein concentrations). Our conclusion, therefore, was that the observed quenching was most likely due to energy transfer from the QDs to the protein by Förster resonance energy transfer (FRET), which would imply binding of the protein to the QDs to form a conjugate between the two.

No significant differences were seen between the quenching brought about by the RCLH1g or RCLH1Xg complexes (Fig. 4(c)), thus indicating that energy transfer was not affected by the structural or optical differences between the two. A lesser extent of quenching (maximum of ∼55%) was achieved using LH1g complexes purified from a green strain lacking RCs, PufX and LH2 (Fig. 4(c), blue), thus showing that the quenching of QD emission was not solely dependent on the presence of RCs.

### 3.5 Binding interactions between QDs and RCLH1(X) complexes

**Fig. 5:**
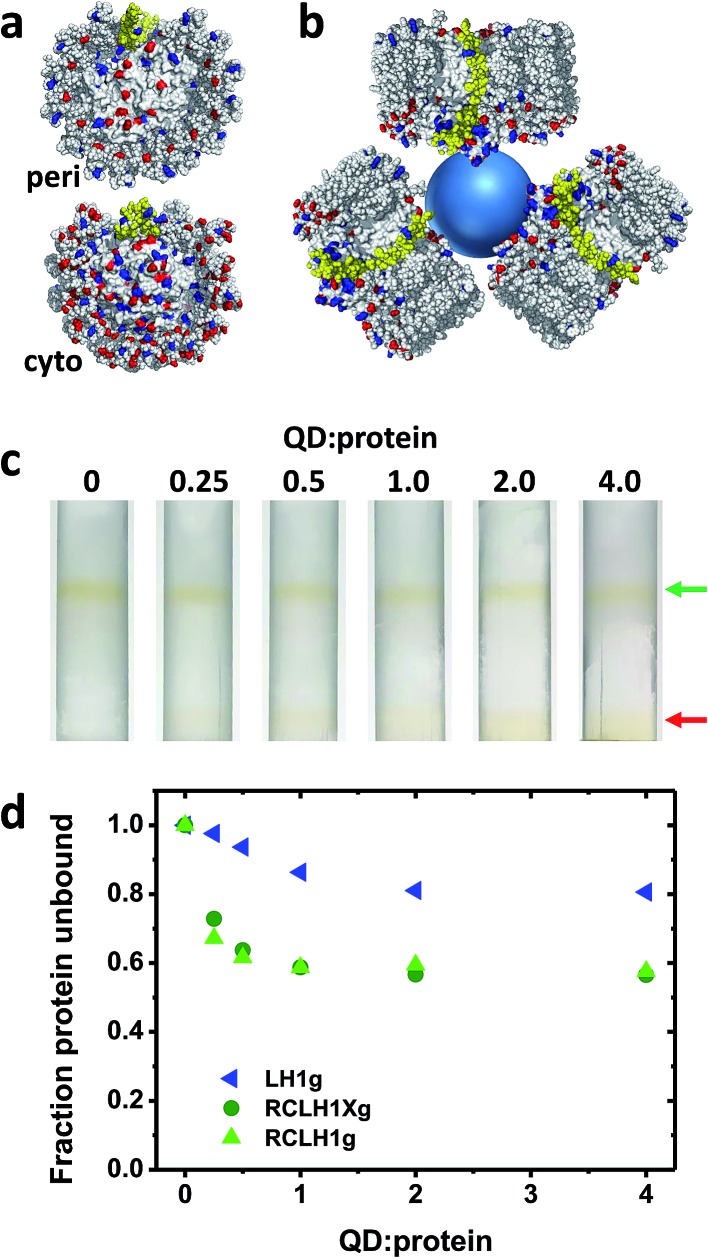
Binding of RCLH1(X) complexes to 6.5 nm CdTe QDs. (a) Distribution of acidic and basic residues on the surface of the *Rba. sphaeroides* RCLH1X complex exposed at the periplasmic (top) and cytoplasmic (bottom) side of the membrane. Glu and Asp residues are shown in red and Lys and Arg residues are shown in blue. PufX is shown in yellow. (b) Schematic of three 12 nm diameter RCLH1X complexes (view parallel to the membrane) positioned around a 6.5 nm diameter QD (blue sphere). (c) Separation of the unbound RCLH1Xg protein (upper band, green arrow) from the RCLH1Xg protein bound to the QDs (lower band, red arrow) on three step sucrose density gradients. (d) Variation with QD : protein ratio of unbound protein as a fraction of the total.

The nature of the conjugates formed between the 6.5 nm diameter CdTe QDs and the RCLH1(X) complexes is speculated upon in Fig. 5(a) and (b). The periplasmic and cytoplasmic surfaces of the RCLH1X complex do not possess a cluster of mostly basic residues that could provide an interaction site for a QD coated with carboxyl groups (Fig. 5(a)). Thus, if binding between RCLH1(X) complexes and QDs is electrostatic, as has been proposed for RCs,^57^ it is not obvious that this would occur at a unique site on the protein surface. Regarding the stoichiometry of protein binding to the QDs, based on the available X-ray crystal structure the diameter of an RCLH1X complex is found to be around 12 nm, which is a little under twice that of a 6.5 nm diameter QD. As a result, as illustrated in Fig. 5(b), it seems unlikely that more than three RCLH1(X) complexes could fit around a single QD (bearing in mind that the true diameter of the former is further extended by a DDM micelle), or two if the binding site is on a recessed area of the protein surface. However, this estimate seems at odds with the finding that between five and ten proteins were needed to maximise the quenching of QD emission (Fig. 4(c)).

To explore this further, “pull-down” assays were carried out using three-step sucrose density gradients in which QDs were titrated against a fixed concentration of protein. The basis of this assay is that RCLH1(X) complexes band at a 0%/30% sucrose interface but QDs band at a lower 30%/60% sucrose interface, with any RCLH1(X) complexes bound to QDs also being pulled down to the lower interface. The upper pigmented band observed after centrifugation therefore comprises free RCLH1(X) complexes (Fig. 5(c), green arrow) whereas the lower band comprises RCLH1(X)/QD conjugates and free QDs (Fig. 5(c), red arrow).

The absorbance spectra of fractions from sets of gradients loaded with RCLH1Xg and RCLH1g complexes showed that, despite the presence of an equivalence or molar excess of QDs in three of the six gradients, the amount of protein binding to the QDs levelled off at around 40% of the total (Fig. 5(d)). The most likely interpretation of this is that the RCLH1(X) complexes exist in two populations, and one of which, the major part, is not able to bind to the QDs. Regardless of the cause, this observation clarified why between five and ten proteins were needed to saturate the quenching of QD emission (Fig. 4(c)), as it implied that the ratio of “competent” proteins was actually between two and four per QD, which is in better accordance with the expected nature of a protein–QD conjugate given the sizes of the two components (Fig. 5(b)). The same analysis with the LH1g complexes revealed weaker binding, levelling off at around 20% of the total protein. By the same rationale this would equate to one or two LH1 per QD, which is lower than for the RCLH1(X)g complexes but consistent with the observed weaker quenching. LH1g titration confirmed the inference from the emission quenching experiments that the binding of RCLH1(X) complexes to QDs was not solely mediated by the RC component.

### 3.6 How does the removal of PufX affect protein stability?

**Fig. 6:**
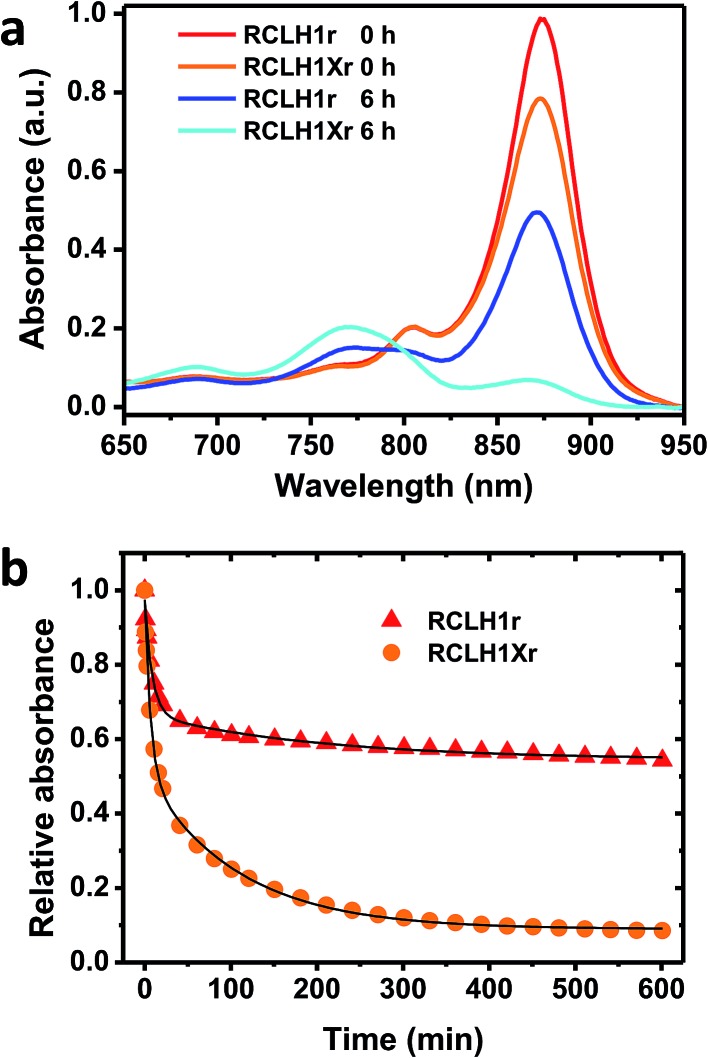
Thermal stabilities of the RCLH1Xr and RCLH1r proteins. (a) Near-IR absorbance spectra of the pigment-protein solutions before and after heating at 60 °C for six hours, (b) decay of the amplitude of the Q_*y*_ absorbance band of the LH1 BChls over a 10 hour incubation at 60 °C. The data were fitted with two exponential terms and an offset to illustrate their biphasic nature.

The possible effects of removal of PufX on the stability of the RCLH1 complex were addressed by repeated absorbance scanning of protein solutions incubated at 60 °C (Fig. 6). For both the RCLH1Xr and RCLH1r complexes, heating produced a decrease of the main absorbance band due to the unfolding of LH1 and release of BChl; the sample spectra in Fig. 6(a) show how the absorbance changes over the first six hours. There was also a drop in the smaller absorbance band at 805 nm, indicating the simultaneous unfolding of the RC, and the appearance of absorbance at 770 nm due to free BChl. The extent of this loss of the native spectrum was markedly greater for the native RCLH1Xr complex (Fig. 6(b), orange to cyan) than for the engineered RCLH1r complex (Fig. 6(a), red to blue). The time dependence of this diagnostic decrease in the LH1 Q_*y*_ band during incubation at 60 °C (Fig. 6(b)) revealed that the engineering of a closed ring of LH1 protein was associated with a significant increase in the stability of the pigment-protein. Although the absolute kinetics varied, this enhanced stability was seen over the range of 30 °C to 70 °C (data not shown), and for equivalent complexes with green carotenoids (data not shown).

As can be seen from Fig. 6(b) the decay of native LH1 absorbance was biphasic over the period monitored, with a satisfactory fit requiring a two-exponential function with time constants of <10 minutes and >100 minutes. The significance of this is not yet clear, but could again indicate two populations of RCLH1(X) complexes, in this case a minor population that is very sensitive to unfolding in response to heat stress and a major population that is more resistant, and whose resistance is markedly enhanced through the removal of PufX.

### 3.7 Why are PufX-minus mutants non-photosynthetic?

**Fig. 7:**
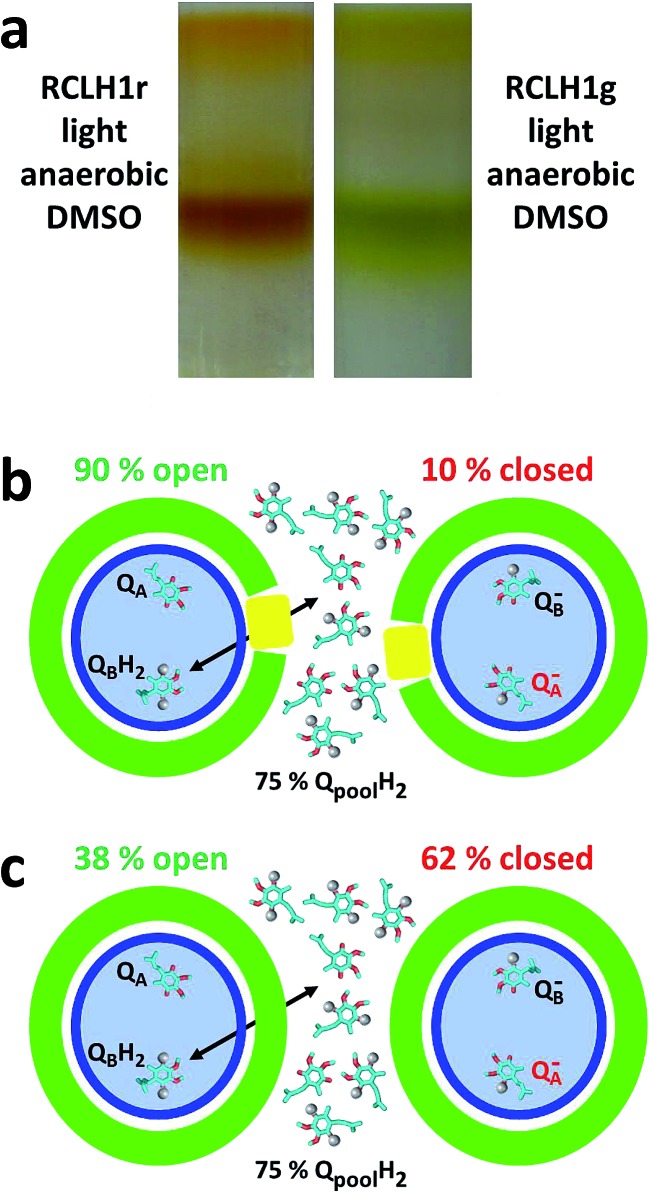
Proteins from cells grown in DMSO and the cause of the phenotype of PufX-minus strains. (a) Monomeric RCLH1 complexes from cells grown under light/anaerobic conditions in the presence of DMSO. (b) In native RCLH1X complexes, a 75% reduction of the intramembrane quinone pool results in 10% of the RCs being closed due to Q_A_ being reduced (the electrons are represented as grey spheres). (c) In engineered RCLH1 complexes, 62% of the RCs are closed under the same conditions, but quinone exchange with the intramembrane pool is not prevented.

The recent description of the location and gross conformation of PufX^22^ supports the conclusions arrived at previously through research on PufX-deficient *Rba. sphaeroides* and *Rba. capsulatus* mutants that one role of this protein is to prevent closure of the ring of LH1 that surrounds the RC (see ref. 31 for a review). A commonly-held view is that, in doing this, PufX enables the exchange of reduced and oxidised quinone between the RC Q_B_ site and the intra-membrane quinone pool, and that the removal of PufX blocks this exchange, leading to a photosynthesis-minus phenotype (see ref. 38 for a recent discussion). However, two lines of evidence argue against this interpretation. First, a detailed examination of electron transfer involving RCLH1X and RCLH1 complexes in photosynthetic membranes by Comayras and co-workers showed that, although it is slowed by a factor of two, transfer of Q_10_H_2_ from the RC to the cyt *bc*_1_ complex still takes place in the absence of PufX.^58^ The slowing was attributed to a change in membrane organisation, with the removal of PufX actually delaying the release of Q_10_H_2_ from the Q_B_ site by only ∼1 ms.^58^ Second, anaerobic/photosynthetic growth can be restored to a PufX-deficient strain of *Rba. sphaeroides* by supplementation of the growth medium with dimethyl sulphoxide (DMSO), which acts as an oxidant for the quinone pool through the action of DMSO reductase.^59^–^61^ To validate this second (often overlooked) observation using the strains that provided the proteins for our photocurrent measurements, in the present work we established that strains RCLH1r and RCLH1g would grow under photosynthetic conditions in the presence of 20 mM DMSO. Cells grown in this way yielded exclusively monomeric RCLH1 complexes on sucrose density gradients (Fig. 7(a)) that exhibited the elevated LH1 absorbance at ∼873 nm relative to RC absorbance at 805 nm, which is diagnostic of the absence of PufX (data not shown).

If these two lines of evidence imply that quinone diffusion between the RC and intramembrane pool is not actually blocked in the absence of PufX, why do PufX-minus mutants not grow under standard photosynthetic conditions? From their study of PufX-deficient membranes, Comayras and co-workers concluded that the likely cause is a change to the structure of the RCLH1 complex that modifies the properties of the Q_B_ ubiquinone.^58^ On the basis of their spectroscopic data they proposed that this change, as-yet not identified, stabilises Q_B_^–^ and lowers the equilibrium constant for the second electron transfer reaction Q_A_^–^Q_B_^–^ → Q_A_Q_B_H_2_. As a result, Q_A_^–^ is accumulated to a greater extent than normal under the reducing conditions prevalent in an anaerobic photosynthetic culture, thus “closing” the RCs and shutting down cyclic electron transfer. Experimental evidence supporting this mechanism has been provided by Stahl and co-workers^62^ who used ultrafast infrared spectroscopy to show that, under identical reducing conditions, charge separation in RCLH1Xr complexes forms P^+^Q_A_^–^ but charge separation in RCLH1r complexes stops at P^+^H_A_^–^, thus implying that Q_A_ is pre-reduced in the absence of PufX.

In their 2005 report, Comayras and co-workers determined the percentage of Q_A_ quinones that are reduced at any given redox state of the intramembrane quinone pool in the presence and absence of PufX (see Fig. 9 in ref. 58). Data from that plot are incorporated into the schemes in Fig. 7(b) and (c), which illustrate the mechanism Comayras and co-workers proposed to account for the consequences of removal of PufX. The schemes show the locked-in Q_A_ and mobile Q_B_ quinone in the RC (blue ellipse), and the intramembrane pool with which the quinone at Q_B_ can exchange by passing through the surrounding LH1/PufX ring (green/yellow). When 75% of the quinones in the intramembrane pool are reduced (electrons represented as grey spheres) only 10% of the RCs in the RCLH1X complexes are closed (Fig. 7(b)) because the reaction Q_A_^–^Q_B_^–^ → Q_A_Q_B_H_2_ favours the product. In contrast, due to the destabilisation of Q_B_^–^ and a shift in the equilibrium for this reaction, 62% of the RCs are closed in the RCLH1(X) complexes under the same conditions (Fig. 7(c)). This mechanism provides an explanation for the inability of PufX-deficient strains to grow under standard photosynthetic conditions despite the fact that quinone diffusion between the Q_B_ site and the (largely reduced) intramembrane pool is not prevented by the removal of PufX. The effect of DMSO can be explained by redox poising,^63^,^64^ with the oxidant draining electrons from the quinone pool and hence the Q_B_ and Q_A_ sites. This lowers the percentage of RCs with a reduced Q_A_ and so opens them for charge separation. It is known that such redox poising by an auxiliary oxidant is required for anaerobic/photoheterotrophic growth of purple bacteria on strongly reducing carbon sources such as butyrate,^63^ and this phenomenon has also been demonstrated *in vitro* using flash spectroscopy.^64^ In addition, ultrafast infrared spectroscopy has been used to show that the formation of P^+^Q_A_^–^ by charge separation is restored in RCLH1 complexes upon the addition of DMSO, thus demonstrating its role in opening RCs by alleviating pre-reduction of Q_A_.^62^

This explanation for the inability of PufX-deficient strains to grow under standard photosynthetic conditions also explains why the RCLH1Xr and RCLH1r complexes compared in this report were equally able to support a photocurrent. The dysfunction associated with the absence of PufX should only manifest under strongly reducing conditions, and so has no impact if such conditions are avoided in a photoelectrochemical cell. We therefore conclude that the lower photocurrents published to date for RCLH1 complexes in comparison to those for RCLH1X complexes are due to the particular electrode materials, electrolytes and measuring conditions used in the different studies, rather than being due to an inherent problem with the ability of RCLH1 complexes to support a continuous flux of electrons.

### 3.8 Enhancement of structural stability in a sub-population of RCLH1 complexes

Given that mutated protein complexes are often less structurally-stable than their native counterparts, an unexpected outcome of our comparison of RCLH1X and RCLH1 complexes was the enhanced thermal stability displayed by the latter. The origins of this are as yet unclear, but presumably relate to the assembly of a closed ring of LH1 pigment-protein around the RC rather than the open structure dictated by PufX. Therefore, in addition to a higher light harvesting capacity, another potential benefit of using PufX-deficient RCLH1 complexes for research on bio-photoelectrochemical cells could be the enhanced stability.

Based on the kinetics of LH1 unfolding shown in Fig. 6(b) it seems likely that there is structural heterogeneity within the populations of the purified RCLH1 and RCLH1X complexes. In both cases, loss of the native absorbance spectrum due to thermal unfolding was biphasic, markedly so in the case of the RCLH1 complex, with a sub-population that is apparently unable to tolerate high temperatures for more than a few minutes and a sub-population that is substantially more tolerant. This difference was observed across a range of temperatures and with either red or green carotenoids present. An investigation of the quenching of QD emission by the RCLH1X and RCLH1 complexes also demonstrated evidence of structural heterogeneity, with only around 40% of either the RCLH1X or RCLH1 complexes being able to bind to an excess of QDs. The basis of this structural heterogeneity is the subject of ongoing investigations, alongside an exploration of methods for physically separating the apparently less stable sub-population of RCLH1 complexes from the sub-population that seems to exhibit a strongly enhanced stability compared to that of its RCLH1X counterpart. An important consideration in the future development of biohybrid photoelectrochemical cells is the stability of their output, and it seems feasible that substantial improvements in stability could be achieved through the sort of protein engineering described here or through the use of protein/material combinations that enhance the robustness of the protein in a device setting.^51^,^65^

## 4. Conclusions

The purpose of this work was to address concerns that PufX-deficient RCLH1 complexes from photosynthetically-incompetent bacteria may not be suitable as photovoltaic materials for incorporation into biohybrid photoelectrochemical cells because they exhibit a dysfunction that limits the photocurrent densities achievable. The finding that RCLH1 and RCLH1X proteins are equally effective in supporting photocurrents in otherwise identical photoelectrochemical cells provides reassurance that this is not the case. Alongside increasing the light harvesting capacity of the RCLH1 complex, we also indicate that the removal of PufX and enlargement of the LH1 pigment-protein ring confers enhanced structural stability upon the RCLH1 complex that may be beneficial for the future use of these proteins in technological applications.

## Conflicts of interest

There are no conflicts of interest to declare.
